# Insights into the role of Val45 and Gln182 of *Escherichia coli *MutY in DNA substrate binding and specificity

**DOI:** 10.1186/1471-2091-10-19

**Published:** 2009-06-12

**Authors:** Po-Wen Chang, Amrita Madabushi, A-Lien Lu

**Affiliations:** 1 Department of Biochemistry and Molecular Biology, School of Medicine, University of Maryland, 108 North Greene Street, Baltimore, MD 21201, USA

## Abstract

**Background:**

*Escherichia coli *MutY (EcMutY) reduces mutagenesis by removing adenines paired with guanines or 7,8-dihydro-8-oxo-guanines (8-oxoG). V45 and Q182 of EcMutY are considered to be the key determinants of adenine specificity. Both residues are spatially close to each other in the active site and are conserved in MutY family proteins but not in *Methanobacterium thermoautotrophicum *Mig.*MthI *T/G mismatch DNA glycosylase (A50 and L187 at the corresponding respective positions).

**Results:**

Targeted mutagenesis study was performed to determine the substrate specificities of V45A, Q182L, and V45A/Q182L double mutant of EcMutY. All three mutants had significantly lower binding and glycosylase activities for A/G and A/8-oxoG mismatches than the wild-type enzyme. The double mutant exhibited an additive reduction in binding to both the A/G and A/GO in comparison to the single mutants. These mutants were also tested for binding and glycosylase activities with T/G- or T/8-oxoG-containing DNA. Both V45A and Q182L mutants had substantially increased affinities towards T/G, however, they did not exhibit any T/G or T/8-oxoG glycosylase activity. Surprisingly, the V45A/Q182L double mutant had similar binding affinities to T/G as the wild-type EcMutY. V45A, Q182L, and V45A/Q182L EcMutY mutants could not reduce the G:C to T:A mutation frequency of a *mutY *mutant. Expression of the V45A mutant protein caused a dominant negative phenotype with an increased G:C to A:T mutation frequency.

**Conclusion:**

The substrate specificities are altered in V45A, Q182L, and V45A/Q182L EcMutY mutants. V45A and Q182L mutants had reduced binding and glycosylase activities for A/G and A/8-oxoG mismatches and increased affinities towards T/G mismatch. However, in contrast to a previous report that Mig.*MthI *thymine DNA glycosylase can be converted to a MutY-like adenine glycosylase by replacing two residues (A50V and L187Q), both V45A and Q182L EcMutY mutants did not exhibit any T/G or T/8-oxoG glycosylase activity. The dominant negative phenotype of V45A EcMutY mutant protein is probably caused by its increased binding affinity to T/G mismatch and thus inhibiting other repair pathways.

## Background

Reactive oxygen species are generated by endogenous processes such as mitochondrial oxidative phosphorylation as well as exogenously following exposure to ionizing radiation and chemicals [1]. Oxidative DNA damage including strand breaks and oxidative base lesions are specifically repaired by base excision repair pathways [2]. The first step in this pathway is carried out by a lesion-specific DNA glycosylase, which cleaves the N-glycosidic bond between the base and deoxyribose sugar [3]. The most abundant and highly mutagenic oxidative DNA damage lesion is 8-oxo-7,8-dihydroguanine (8-oxo-G or GO) that can form base-pair with adenine or cytosine during DNA replication to produce a G:C to T:A transversion [4,5]. In *Escherichia coli*, MutT, MutM (Fpg), MutY, MutS, and Nei (End VIII) are involved in defending against the mutagenic effects of 8-oxoG lesions [reviewed in [6] and [4]. The MutT protein has pyrophosphohydrolase activity, which eliminates 8-oxo-dGTP from the nucleotide pool. MutM glycosylase (Fpg protein) removes both mutagenic GO adducts and ring-opened purine lesions paired with cytosines. MutS and MutY increase replication fidelity by removing the adenines misincorporated opposite GO or G [5,7,8], and thus reduce G:C to T:A transversions. Nei can excise GO, when GO is paired opposite a cytosine or adenine and can serve as a backup pathway to repair 8-oxoG in the absence of MutM and MutY [6,9].

**Figure 1:**
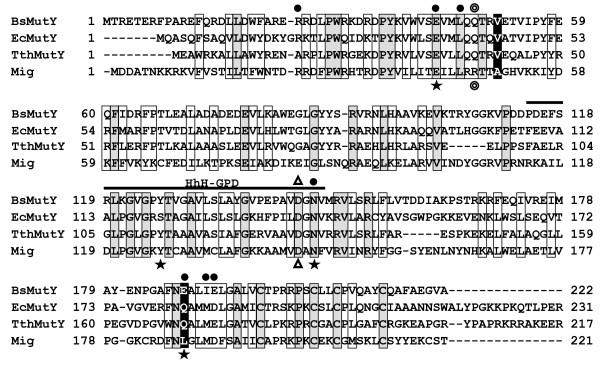
**Sequence Alignment**. The protein sequences of *B. stearothermophilus *MutY (1VRLA, BsMutY), *E. coli *MutY (AP 003518, EcMutY), *T. thermophilus *MutY (YP 145164, TthMutY), and *M. thermoautotrophicum *Mig.*Mth*I (P 29588, Mig) were aligned using ClustalW algorithm. The open and shadowed boxes represent conserved and identical residues respectively. The two residues subjected to mutation in this study are shaded in black. The highly conserved HhH-GPD motif is indicated. Filled circles indicate EcMutY residues involved in adenine binding and stars indicate Mig.*Mth*1 residues important for thymine recognition. The hollow circle and triangle mark the base flipper and the conserved catalytic Asp, respectively.

Unlike most DNA glycosylases, MutY does not target a damaged base, but a normal base, e.g., removing A mispaired with GO, C, and 5-hydroxyuracil [reviewed in [4]]. In addition, MutY can excise guanines from G/GO mismatches [5]. MutY also binds tightly to DNA containing T/GO and C/GO, however, it exhibits no catalytic activity on them [10]. The N-terminal domain of EcMutY retains the catalytic activity [10-14] while the C-terminal domain of MutY plays an important role in the recognition of GO lesions [10,11,14,15]. The X-ray crystal structures of EcMutY catalytic domain with bound adenine [16] and intact *Bacillus stearothermophilus *MutY (Bs-MutY) bound to DNA [17] show that MutY distorts the bound DNA substrate and the mismatched A is flipped out of the helix. In the active site pocket of EcMutY, seven amino acids (R19, E37, L40, N140, Q182, M185, and D186) are involved in adenine binding (filled circles in Figure 1) [16]. V45 and Q182 of EcMutY are proposed to be key residues in determining substrate specificity. V45 is considered to be important for glycosylase activity because V45N is inactive [16] and is conserved among MutY family members (Figure 1). Q182 forms a hydrogen bond with adenine at the domain interface of EcMutY, but is less conserved [18].

The N-terminal domain of MutY shares similar structure, including the helix-hairpin-helix (HhH) and Gly/Pro Asp loop motifs (Figure 1), with AlkA, EndoIII, and OGG1. The G/T mismatch glycosylase Mig.*Mth*I of *Methanobacterium thermoautotrophicim *is a member of the HhH superfamily [19] and is involved in reducing spontaneous deaminated bases from 5-methyl-cytosine residues. The substrate specificity of Mig.*Mth*I is in the order: U/G = T/G > G/G > T/C = U/C > A/G. In the binding pocket for the target base, Mig.*Mth*I shares six out of seven residues of EcMutY's adenine binding (R19, E37, L40, N140, Q182, M185, and D186). The only non-conserved residue in this binding pocket is Q182 of EcMutY, which corresponds to L187 of Mig.*Mth*I (Figure 1). Interestingly, Fondufe-Mittendorf *et al. *[20] could convert Mig.*Mth*I into a MutY-like glycosylase with altered substrate preference of Mig.*Mth*I from T/G to A/G by replacing two residues (A50V and L187Q) in the substrate binding pocket. Their data point to the potential importance of V45 and Q182 in EcMutY substrate recognition.

In this study, we have investigated the role of V45 and Q182 of EcMutY by targeted mutagenesis. V45A, Q182L, and V45A/Q182L mutant proteins have reduced binding affinity and glycosylase activity to DNA containing A/G or A/GO mismatches. These mutants are impaired to complement *E. coli mutY *mutants *in vivo*. The affinities to T/G dramatically increased in V45A and Q182L mutants, but not in the V45A/Q182L double mutant. However, V45A, Q182L, and V45A/Q182L mutant proteins do not exhibit glycosylase activity towards T/G or T/GO. Therefore, V45 and Q182 of EcMutY are important for the adenine-specific activity of EcMutY.

## Results and discussion

### Selection of EcMutY mutation

**Figure 2:**
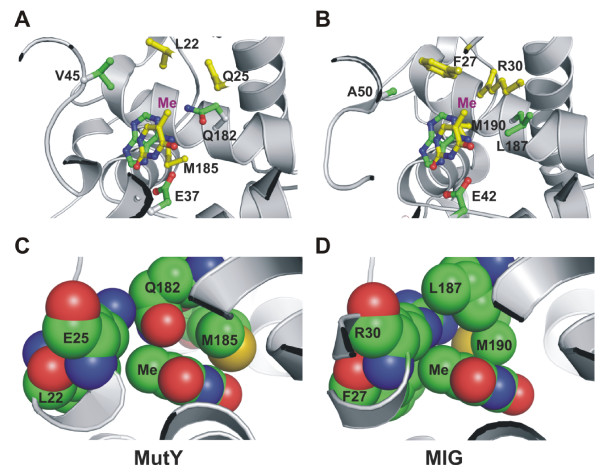
**(A). *E. coli *MutY structure bound to adenine base (RCSB ID 1MUD) with thymine docked into the binding pocket as proposed by Mol *et al. ***[23]** for Mig.MthI**. The residues of EcMutY known to be important for adenine recognition (E37, V45, and Q182) and the adenine and thymine bases are colored by atom type except the carbons in the docked thymine are colored yellow. Residues homologous to those residues in Mig.MthI proposed to be important in forming a hydrophobic pocket around the thymine methyl group (marked Me) are colored yellow. (B). The active site of Mig.MthI with thymine docked in according to the proposed orientation and hydrogen-bonding scheme [23]. The adenine from the EcMutY structure is included as a reference to show the relative position of key residues in the active site. As in (A), residues implicated in solvating the thymine methyl group are colored yellow. Y126, which is proposed to form a hydrogen bond with O2 of thymine is not pictured for reasons of clarity. (C). A space-filling model of thymine docked into the EcMutY active site, with an emphasis on interactions with the methyl group (Me). (D). A space-filling model of thymine docked into the Mig.MthI active site as proposed [23].

The study by Fondufe-Mittendorf *et al*. [20] indicates that the substrate preference of Mig.*Mth*I can be altered from T/G to A/G by replacing two residues (A50V and L187Q) in the substrate binding pocket. Their results are in agreement that V45 and Q182 of EcMutY are the key determinants of adenine specificity [16]. According to the adenine soaked EcMutY structure (Figure 2A) [16], V45 is located in the minor-groove reading α2–α3 motif (37-EVMLQQTQV-45) and Q182 is located in the adenine recognition α10 motif (182-QAMMD-186). Interestingly, the proximity of V45 and Q182 to adenine in EcMutY structure closely correlates with the BsMutY structure with the DNA product and cleaved adenine [17]. However, the residues corresponding to Val45 and Gln182 in D144N BsMutY with A/GO-containing DNA (so called the lesion recognition complex) [17] do not directly make contacts with the adenine of the A/GO mismatch. In the later structure, the Val seems to be positioned below the adenine, but the Glu188 of BsMutY (corresponding to Glu182 of EcMutY) is far away from adenine. The fact that there are differences in adenine recognition among various MutY structures raises a question whether these residues really are involved directly in adenine recognition. To determine whether these two residues are important for adenine recognition and excision, we carried out targeted mutagenesis study to determine the effect of EcMutY V45A, Q182L, and double mutant on repair of A/G- and A/GO-containing DNA. We also further determined whether their substrate specificity is altered in terms of binding or catalysis of T/G or T/GO mismatches.

### A/G and A/GO binding and glycosylase activity

**Figure 3:**
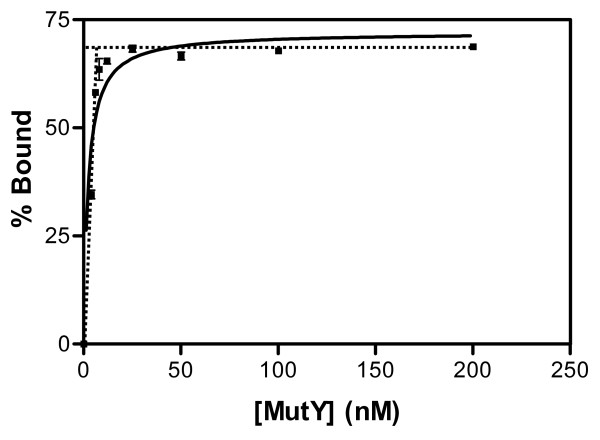
**Active site titration of wild-type EcMutY by binding to A/GO-containing DNA**. DNA binding was assayed with 8 nM of A/GO-containing DNA and 2 to 200 nM (as measured by Bradford assay) of MutY. The result shows the average for two independent experiments. Calculated percentage bound were plotted as a function of EcMutY concentration and fitted by a modified version of Eq. 2 except [P]_active _is replaced with protein concentration determined by Bradford assay. The increase in wild-type (WT) EcMutY binding reached a maximum at a concentration about 20 nM of MutY and 5.44 nM DNA was bound (68% of 8 nM). This line (horizontal dotted line) intercepts with the slope line of initial rate (slant dotted line) at 8 nM of EcMutY. Thus, the WT EcMutY preparation is 68% active.

We compared the binding affinities of V45A, Q182L, and V45A/Q182L double mutants with wild-type EcMutY to DNA substrates containing A/G and A/GO mismatches. First, we determined the active site concentrations of these proteins by performing A/GO binding with DNA concentrations which are at least 17-fold higher than their estimated dissociation constants (*K*_*d*_). Figure 3 shows the average results from two independent experiments with 8 nM of A/GO-DNA. The increase in wild-type (WT) EcMutY binding reached a maximum at a concentration about 25 nM of MutY and 5.44 nM DNA was bound (68% of 8 nM). This horizontal line intercepts with the slope line of initial rate at 8 nM of MutY. Thus, the WT EcMutY preparation is 68% active. Similar analyses showed that V45A, Q182L, and V45A/Q182L were 48%, 25%, and 40% active, respectively. Dissociation constant value for each mutant was determined with nine enzyme concentrations by at least three independent experiments (Table 1). Because MutY displays a high affinity for its products [21], the *K*_*d *_values measure its affinities to a mixture of the substrate and the product. Compared to wild-type EcMutY of which the *K*_*d *_to A/G is 1.3 ± 0.11 nM, the V45A and Q182L mutants had *K*_*d *_values of 6.1 ± 1.7 and 8.9 ± 5.0 nM, respectively. The *K*_*d *_value (15 ± 2 nM) of V45A/Q182L double mutant was higher than the single mutants. Thus, the affinities to A/G of V45A, Q182L, and V45A/Q182L are weakened by 5-, 7-, and 12-fold, respectively. For A/GO binding, V45A and Q182L mutations resulted in a 94- and 11-fold increase in *K*_*d *_values, respectively. The V45A/Q182L double mutation resulted in a 102-fold increase in *K*_*d *_value (i.e. 102-fold weaker binding affinity). Thus, V45A is more defective in A/GO binding than A/G binding as compared to wild-type MutY. It is interesting to note that the double mutant exhibited an additive reduction in binding to both the A/G and A/GO as for the single mutants. These results confirm that V45 and Q182 play important but independent roles in adenine binding.

**Table 1:** Apparent dissociation constants (*K*_*d*_) of MutY mutants.

DNA	WT	V45A	Q182L	V45A/Q182L
A/G	1.3 ± 0.11^a^	6.1 ± 1.7 (0.21)^b^	8.9 ± 5.0 (0.15)	15 ± 2 (0.09)
A/GO	0.0048 ± 0.0029	0.45 ± 0.06 (0.01)	0.051 ± 0.009 (0.09)	0.59 ± 0.12 (0.01)
T/G	14 ± 1	0.011 ± 0.004 (1273)	0.043 ± 0.014 (326)	8.4 ± 0.7 (1.7)
T/GO	0.036 ± 0.013	0.024 ± 0.009 (1.5)	0.088 ± 0.061 (0.41)	0.013 ± 0.008 (2.77)

^a ^*K*_d _values (nM) are mean ± standard deviation for more than three experiments using nine protein concentrations.

^b^The numbers in parenthesis are the folds of binding affinity relative to wild-type (WT) MutY.

**Figure 4:**
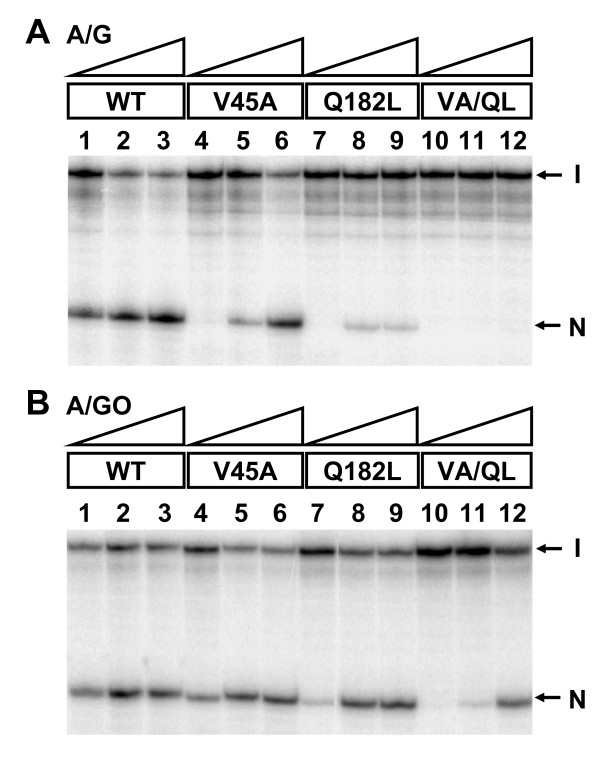
**DNA glycosylase activities of EcMutY mutants**. 1.8 fmol (0.18 nM) of 3'-end labeled A/G- (panel A) or A/GO- (panel B) containing 44-mer oligonucleotides were incubated with three concentrations (1, 10, and 50 nM based on Bradford assay) of EcMutY at 37°C for 30 min. With the active site titration assay upon binding with the A/GO-DNA, the active enzyme concentrations in the experiments are indicated as following. Lanes 1–3, wild-type (WT) EcMutY at 0.68, 6.8, and 34 nM were added. Lanes 4–6, V45A EcMutY at 0.48, 4.8, and 24 nM were added. Lanes 7–9, Q182L EcMutY at 0.25, 2.5, and 12.5 nM were added. Lanes 10–12, V45A/Q182L EcMutY at 0.4, 4, and 20 nM were added. After the reaction, the products were treated with 0.1 M NaOH and heated at 90°C for 30 min, followed by resuspension in formamide dye, and fractionated on a 14% denaturing sequencing gel. Arrows indicate the positions of intact oligonucleotide (I) and the nicked product (N).

Because V45A and Q182L EcMutY mutants showed weakened A/G and A/GO affinities, we examined their glycosylase activities on A/G and A/GO (Figure 4). V45A mutant showed decrease in A/G glycosylase activity at 0.48 and 4.8 nM concentrations (Figure 4A, lanes 4–5) while Q182L mutant showed no activity at 0.25 nM and decrease in activity at 2.5, and 12.5 nM (Figure 4A, lanes 7–9), as compared to wild-type MutY. The glycosylase activity of V45A/Q182L mutant on A/G was nearly undetectable at 20 nM (Figure 4A, lane 12). With A/GO substrate, both V45A and Q182L mutants showed decrease in activity at 0.48 and 0.25 nM, respectively (Figure 4B, lanes 4 and 7), whereas the V45A/Q182L double mutant showed no activity at 0.4 nM and decreased activity at 4, and 20 nM (Figure 4B, lane 10–12). The glycosylase activity assayed at the indicated active site concentrations is based on the active site titration results upon binding with A/GO-DNA. Since the mutants' glycosylase activities are affected differently from DNA binding, thus, the active site concentrations measured by DNA binding may be different from those measured by DNA glycosylase activity.

**Figure 5:**
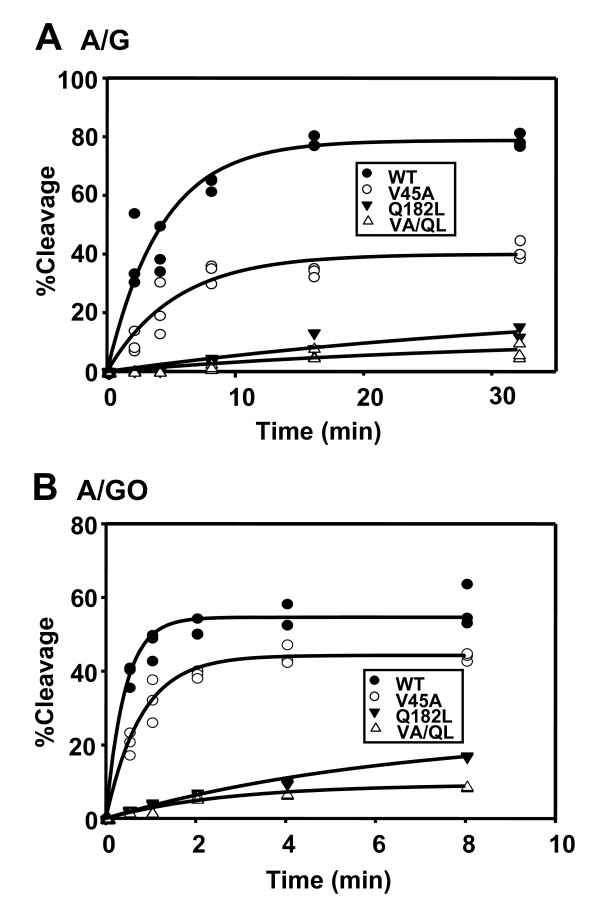
**Time course studies of EcMutY mutant glycosylase activities**. Panel A, A/G-containing DNA substrate (0.18 nM) was incubated with 49, 35, 18, and 29 nM of active site concentrations (all are 72 nM by Bradford assay) of WT, V45A, Q182L, and V45A/Q182L EcMutY, respectively, for various times at 37°C. Panel B is similar to panel A except that A/GO-containing DNA substrate (0.18 nM) was incubated with EcMutY for various times at 4°C. After reaction, the products were immediately frozen at -70°C in the presence of 0.1 N NaOH, heated at 90°C for 30 min, supplemented with 5 μl formamide dye, and loaded onto 14% 7 M urea sequencing gels. Numerical data were obtained from PhosphorImager quantitative analyses of gel images over three experiments. Percentages of DNA cleaved were plotted as a function of time.

We also used single-turnover glycosylase kinetics with enzyme in excess over DNA (time-course study) to compare the activities of wild-type and mutant EcMutY proteins. As shown in Figure 5, the rates of cleavage on both A/G- and A/GO-containing DNA by MutY-V45A were only slightly lower than those of the wild-type EcMutY protein. However, the rates of cleavage of both A/G- and A/GO-containing DNA were much lower for Q182L and V45A/Q182L mutants than those of the wild-type EcMutY (Figure 5). The rate constants (*k*_2_) of Q182L and V45A/Q182L mutants with A/G mismatch at 37°C were 0.025 ± 0.016 Min^-1 ^and 0.032 ± 0.026 Min^-1^, respectively, and were about 10-fold lower that that of the wild-type EcMutY (with *k*_2 _of 0.24 ± 0.03 Min^-1^) (Table 2). The *k*_2 _of Q182L and V45A/Q182L mutants with A/GO mismatch at 4°C were 0.16 ± 0.02 Min^-1 ^and 0.37 ± 0.05 Min^-1^, respectively, and were about 10-fold lower that that of the wild-type EcMutY (with *k*_2 _of 2.4 ± 0.3 Min^-1^) (Table 2). These results indicate that the overall repairing ability is reduced by Q182L and V45A/Q182L mutants but not by the V45A mutant. The above results demonstrate that V45A and Q182L mutations have significant effect on binding and glycosylase activities to A/G and A/GO mispairs. A catalytic domain of EcMutY with a V45N mutation has been shown to be inactive [16]. These results indicate that V45 and Q182 are significant for the adenine-specific activity of EcMutY.

**Table 2:** Rate constants (*K*_2_) of MutY mutants.

DNA	WT	V45A	Q182L	V45A/Q182L
A/G	0.24 ± 0.03^a^	0.19 ± 0.03 (0.80)^b^	0.025 ± 0.016 (0.11)	0.032 ± 0.026 (0.14)
A/GO	2.4 ± 0.3	1.3 ± 0.1 (0.54)	0.16 ± 0.02 (0.07)	0.37 ± 0.05 (0.15)

^a^k_2 _values (Min^-1^) are means ± standard deviation for more than three experiments. k_2 _values with A/G and A/GO are determined at 37°C and 20°C, respectively.

^b ^The numbers in parenthesis are the folds of rate relative to wild-type (WT) MutY.

### T/G and T/GO binding and glycosylase activity

We next measured the T/G and T/GO binding affinities of these mutants. Wild-type EcMutY showed a comparatively high *K*_*d *_(14 ± 1 nM) with a T/G mismatch (Table 1), however, this value is much lower than that of EcMutY with C:G homoduplex (315 ± 49 nM) [22]. The *K*_*d *_values of V45A and Q182L mutants to T/G drastically decreased to 0.011 ± 0.004 nM and 0.043 ± 0.014 nM, representing a 1273 and 326-fold increase in binding affinity, respectively (Table 1). Surprisingly, V45A/Q182L double mutant showed a *K*_*d *_value of 8.4 ± 0.7 nM, which is close to that of wild-type EcMutY. The geometry or architecture of the substrate binding pocket in the V45A/Q182L double mutant may not favor thymine binding. For T/GO binding, V45A, Q182L, and V45A/Q182L mutants showed similar *K*_*d *_values as wild-type EcMutY (Table 1), indicating that the T/GO binding affinity of EcMutY does not involve V45 and Q182. This result is consistent with the findings of Li *et al*. [10] that tight T/GO binding is controlled by the C-terminal domain of EcMutY.

Because Mig.*Mth*I T/G glycosylase can be converted to an A/G glycosylase by replacing two residues (A50V and L187Q) [20], we examined the glycosylase activities of Vl45A, Q182L, and V45A/Q182L on T/G and T/GO. None of the EcMutY mutants showed any T/G and T/GO glycosylase activity at enzyme concentration up to 1 μM under different buffers and temperatures (data now shown). Therefore, recognition and glycosylase of EcMutY mutants to T/G and T/GO are controlled by different mechanisms.

The previous success to mutate Mig.*Mth*I into MutY-like enzyme [20] is in contrast to our failure to covert EcMutY to thymine glycosylase. It is possible that it is easier to catalyze adenine removal than thymine excision. It is interesting to point out that wild-type Mig.*Mth*I is a weak adenine glycosylase and Mig.*Mth*I with A50V/L187Q mutation becomes a stronger adenine glycosylase. However, wild-type EcMutY has no thymine glycosylase activity and V45A/Q182L mutant may be harder to gain this activity. In addition, the active site of Mig.*Mth*I is more relaxed for both purine and pyrimidine excision while MutY is more strict to excise purines. The difference in the base removal by these two enzymes may be contributed by other residues surrounding the active site.

By aligning MutY and Mig.*Mth*I sequences (Figure 1) and inspecting the structures of Mig.*Mth*I [23], EcMutY catalytic domain [16] with docked thymine (Figure 2), we predicted that several residues may contribute to their difference in activities. The thymine docked into the EcMutY active site preserves the proposed edge-on hydrogen bonding interaction with the conserved glutamate (E37, E42 in Mig.MthI) and can support a potential hydrogen bond with a tyrosine in a potential S120Y mutant (Figure 2A). It is clear from Figs. 2C and 2D that the MutY active site poorly accommodates the methyl group of thymine relative to the Mig.MthI active site. In the present study, one important mutation (Q182L) has been created. Based on our observations and the models shown in Figure 2, the subsequent mutations most likely to promote the conversion of MutY into a thymine glycosylase would be Q25R and L22F. These two mutations would complete the methyl-binding pocket and stabilize the interaction with thymine. In addition, an S120Y mutation, which would provide an additional hydrogen bond (i.e. to the O2 of thymine) might further enhance any thymine glycosylase activity resulting from the Q182L, Q25R, and L22F mutations. However, such predictions highlight the difficulty inherent in designing proteins with novel activities. In particular, it has been shown that Y126S mutation of Mig.*Mth*I inactivates T/G mismatch-specific glycosylase activity [23], however, the presence of Tyr in BsMutY indicates that Tyr may not be the only key residue for T/G catalytic activity. Similarly, BsMutY contains R31 to accommodate the methyl group of thymine relative to the Mig.MthI active site, but does not excise thymine.

Recently, a bifuncional MutY-like glycosylase, TthMutY, has been shown to have strong A/GO and G/GO activities, comparatively weak T/GO and A/G activities, and no T/G glycosylase activity [24]. Unlike Mig.MthI, TthMutY and EcMutY contain extra C-terminal domains which facilitate their recognition of the mispaired GO base. V42 and Q170 of TthMutY are identical with the corresponding residues in EcMutY (V45 and Q182) but different from A50 and L187 of Mig.MthI (Figure 1). Although TthMutY has weak T/GO glycosylase activity, its L19 and Q39 residues, which correspond to F27 and R47 of Mig.MthI, respectively, are also conserved in EcMutY and BsMutY. This suggests that F27 and R47 of Mig.MthI may not be absolutely required for T/G or T/GO glycosylase activity. Interestingly, Y112S TthMutY mutant lost the thymine glycosylase activity supporting that Y126 of Mig.*Mth*I may be important for T/G catalytic activity. It remains to be tested whether S120Y, Q25R, and L22F mutants of EcMutY or in combination with V45A and Q182L can gain T/G glycosylase activity.

### V45A, Q182L, and V45A/Q182L EcMutY mutants cannot complement the *mutY* mutation

The reduced binding and glycosylase activities toward A/G and A/GO of these EcMutY mutants led us to check whether they have normal biological activity. We examined the mutation frequency in rifampicin forward-mutation and Lac^+ ^reversion assays. Rifampicin binds to RNA polymerase (encoded by *rpoB *gene) and inhibits its transcriptional function. Mutations in the rifampicin binding site of RNA polymerase results in cells' resistance to rifampicin. In the absence of both EcMutM and EcMutY, a high level of rifampicin-resistant cells was observed (Table 3, left panel, line 2). The *mutMmutY *double mutant offers a more sensitive complementation assay than the *mutY *single mutant. Expression of wild-type EcMutY complemented the *mutMmutY *double mutation (Table 3, left panel, line 4). Expression of EcMutY-V45A, EcMutY-Q182L and EcMutY-V45A/Q182L mutants partially complemented the deficient EcMutM and EcMutY function. The mutation frequencies are 154, 44, and 178 folds higher, respectively, compared to the CC104 wild-type (Table 3, left panel, lines 1, 5–7).

**Table 3:** Mutation frequencies of λDE3-containing *E. coli mutM mutY *mutant strains.

Strain	Mutation Frequency (Rif^R^/10^8 ^cells)	Fold	Mutation frequency (Lac^+^/10^8 ^cells)	Fold
		
1. CC104 (WT)	4.1 ± 1.3	1	1.2 ± 1.0	1
2. CC104 *mutMmutY*	1464 ± 314	357	486 ± 65	408
3. CC104*M*^-^*Y*^- ^+ pET11a^a^	2055 ± 704	501	156 ± 102	130
4. CC104*M*^-^*Y*^- ^+ pET-MutY^b^	8.8 ± 4.4	2	0.9 ± 0.8	0.8
5. CC104*M*^-^*Y*^- ^+ pET-V45A^c^	630 ± 177	154	6.3 ± 2.1	5.3
6. CC104*M*^-^*Y*^- ^+ pET-Q182L^d^	181 ± 74	44	10.2 ± 8.7	8.5
7. CC104*M*^-^*Y*^- ^+ pET-V45A/Q182L^e^	728 ± 358	178	20 ± 13	16.7

^a ^The strain CC104 mutM mutY contains pET11a which is the expression vector for wild-type and mutant MutY expression. ^b-e ^The strain CC104 mutM mutY expresses indicated MutY protein cloned in the pET11a vector.

CC104 strain was designed to screen G:C→T:A transversions at an essential residue in the active site of β-galactosidase encoded by *lac*Z gene, thus it allowed us to measure this type of mutation in EcMutY-V45A, EcMutY-Q182L and EcMutY-V45A/Q182L mutants. Similar to the results of rifampicin forward assay, the mutation frequency of the *mutMmutY *double mutant increased 400-fold over the wild-type cells (Table 3, right panel, lines 1 and 2). The EcMutY-V45A, EcMutY-Q182L and EcMutY-V45A/Q182L mutants showed partial defect in preventing G:C→T:A transversions as compared to the wild-type EcMutY. Strains expressing V45A, Q182L and V45A/Q182L mutants, respectively, showed 5, 8, and 17- fold higher mutation frequencies than CC104 wild-type (Table 3, right panel, lines 5–7). These increased G:C→T:A mutation frequencies are in agreement with the reduced glycosylase activities with A/G and A/GO mismatches in these mutants.

### Wild-type cells expressing the V45A mutant protein have an increased G:C to A:T mutation frequency

For the strains expressing V45A MutY protein, the G:C→T:A mutation frequency measured by Lac^+ ^ reversion assay was much lower than that measured by the rifampicin forward-mutation assay (154 fold increase in rifampicin forward-mutation assay vs. 5.3-fold increase in the Lac^+^reversion assay) (Table 3, compare lines 5 in right and left panels). Because V45A mutant exhibits milder defect in adenine glycosylase activity than the other two mutants, we suspected that the V45A mutant with a 1273-fold increased T/G affinity (Table 1) might prevent T/G mismatches from being repaired by other DNA repair pathways. To test this idea, we isolated genomic DNA from rifampicin resistant colonies from CC102 (*mutM*^+ ^*mutY*^+^) cells expressing EcMutY-V45A and identified base-base mutations of *rpoB *gene [25]. A significant increase in G:C→A:T transition was found in cells expressing EcMutY-V45A as compared to cells with vector alone (Table 4). For unknown reason, the mutation frequency of A:T to T:A was quite high in CC102 harboring vector and EcMutY-V45A. CC102 strain was designed to screen G:C→A:T transitions in *lac*Z gene. Thus, we further measured the G:C→A:T transition frequency by Lac^+ ^reversion assay in CC102 (*mutM*^+ ^*mutY*^+^) strain expressing EcMutY-V45A. The EcMutY-V45A expressing strain showed a 3.6-fold higher Lac^+ ^reversion frequency. A T-test of two sets of data of rows 1 and 3 in Table 5 shows that this increase is statistically significant with *P *= 0.001 (Table 5). These data are consistent with our postulation that EcMutY-V45A protein acts as a dominant-inhibitor of other DNA repair pathways.

**Table 4:** Mutation distribution of *rpoB *in λDE3-containing *E. coli *CC102 (*mutM*^+^*mutY*^+^) harboring pET11a and pET11a-V45A.

Plasmid	No. of clones with *rpoB *mutation (%)	
	
	pET11a	pET11a-V45A
A:T → T:A	30 (81.1)^a^	19 (61.3)
G:C → A:T	5 (13.5)	12 (38.7)
G:C → T:A	1 (2.7)	0 (0)
A:T → G:C	1 (2.7)	0 (0)
Total	37	31

^a^The number in parenthesis are the percentages of each mutation type.

**Table 5:** Lac^+ ^reversion rate of λDE3-containing *E. coli *CC102 (*mutM*^+^*mutY*^+^) strains

	Mutation frequency (Lac^+^/10^8 ^cells)	Fold
1. CC102 + pET11a	0.45 ± 0.12	1
2. CC102 + pET-MutY	0.51 ± 0.16	1.1
3. CC102 + pET-V45A	1.60 ± 0.36	3.6

## Conclusion

The substrate specificity of DNA glycosylases is very subtle to the change of amino acids located in the DNA binding pocket, and a single amino acid alteration may significantly alter its enzyme activity. Our results show that V45 and Q182 of EcMutY are important for the binding affinity and glycosylase activity to DNA containing A/G or A/GO mismatches. The V45A/Q182L double mutant exhibited an additive reduction in binding to both the A/G and A/GO as for the single mutants. Our unexpected results that EcMutY-V45A and EcMutY-Q182L show increased T/G binding affinity without gaining T/G glycosylase indicate the complicated nature of the DNA-enzyme interaction.

## Methods

### Bacteria strains

*Escherichia coli *DH5α (F^-^, φ*80*-*dlacZΔM15*, *endA1*, *recA1*, *hsdR1*, (r_k_^-^m_k_^+^), *supE*44, *thi*-1, *gry*A96(Nal^r^), *rel*A1, Δ (*lacZYA-argF*) U169) was purchased from Invitrogen. PR70 (Su^- ^sm^r^*lacZ X74 galU galK miA68::*Tn*10*kan) was obtained from M. S. Fox. The *miA68::*Tn*10*kan allele in PR70 contains a transposon at the *mutY *gene at nucleotide 747 and produces a truncated MutY protein [10]. CC102 [*ara *Δ (*lac-proB*)_XIII _*thi *F'-*lacI378 lacZ461 proA*^+^*B*^+^) [26] and CC104, which is identical to CC102 except for the mutation at residue 461 of β-galactosidase, were generous gifts from J. H. Miller. The derivative CC104 *mutM mutY*: *mutM*::mini-kan *mutY*::mini-Tn10 was also from J. H. Miller. λDE3 lysogenic strains were constructed according to the procedures described by Invitrogen. XL1-Blue (*recA1 endA1 gyrA96 thi-1 hsdR17 supE44 relA1 lac *[F *proAB lacI*, *Z*Δ*M15 *Tn*10 *(Tet)]) was purchased from Stratagene.

### Construction of *E. coli mutY* mutants

The mutant *mutY *genes encoding proteins containing V45A, Q182L and V45A/Q182L were constructed by the QuickChange Site-Directed Mutagenesis Kit (Strategene). The mutagenesis PCR primers for the desired amino acid substitutions are listed below.

Chang564 5'-ATGTTGCAACAAACTCAAGCTGCGACCGTTATCCCC-3'

Chang565 5'-GGGGATAACGGTCGCTGCTTGAGTTTGTTGCAACAT-3'

Chang566 5'-GTGGAACGGTTTAATCTGGCCATGATGGATTTGGGTGCG-3'

Chang567 5'-CGCACCCAAATCCATCATGGCCAGATTAAACCGTTCCAC-3'

Primers, Chang564 and Chang565, are designed for the V45A mutant and Chang566 and Chang567 are designed for the Q182L mutant using plasmid pMYW-1 [27] as the template. The plasmids containing the *mutY *mutant genes with V45A or Q182L mutation were named pMY-V45A and pMY-Q182L. The double mutant was derived from pMY-V45A using primers Chang566 and Chang 567. After the PCR reaction, the template plasmids were digested with DpnI restriction and transformed into XL1-Blue supercompetent cells (Strategene). The correct clones were confirmed by DNA sequencing.

### Measurement of mutation frequency

Overnight cultures (0.1 ml) of each strain were plated on LB agar plates containing 0.1 mg/ml rifampicin. The cell titer of each culture was determined by plating 0.1 ml of and 10^-6 ^dilution onto LB agar plates. For each measurement, four independent cultures were plated, and the experiments were repeated at least three times. The mutation frequency was determined by calculating the ratio of Rif^r ^cells to total cells. For LacZ^+ ^reversion mutation assay, overnight cultures (0.2 ml) of each strain were plated on M9 agar plates containing 0.2% lactose and colonies were scored after three days. The ratio of LacZ^+ ^cells to total CC104 cells was calculated to be the G:C→T:A transversion frequency. The ratio of LacZ^+ ^cells to total CC102 cells was calculated to be the G:C→A:T transition frequency.

### EcMutY protein expression and purification

*E. coli *strains PR70/DE3 harboring expression plasmids pMY-V45A, pMY-Q182L, and pMY-V45A/Q182L were grown in LB broth containing 50 μg/ml ampicillin at 37°C. At OD_600 _of 0.6, isopropyl β-D-thiogalactoside (IPTG) was added to the culture to a final concentration of 0.2 mM, and the culture was incubated at 20°C for 16 hrs. The EcMutY mutant proteins were purified by ammonium sulfate precipitation, phosphocellulose, hydroxylapatite, heparin, Hitrap-S column chromatographies as previously described for the wild-type MutY [21]. The purified proteins were divided into small aliquots and stored at -80°C. Protein concentration was determined by Bradford Method.

### Oligonucleotide substrates

The nucleotide sequences of 40-mer DNA substrates containing mismatches used in this study were:

5'-AATTGGGCTCCTCGAGGAATT**X**GCCTTCTGCAGGCATGCC-3'

 3'-CCCGAGGAGCTCCTTAA**Y**CGGAAGACGTCCGTACGGGGCC-5'

(where X = A or T and Y = G or GO). The X-strand was labeled by [γ-^32^p]ATP on the 5' end and then annealed with the Y-strand. The annealed double-stranded oligonucleotides were converted to 44-mers by filling the sticky ends on both sides with Klenow fragment [28].

### EcMutY binding and glycosylase assays

The EcMutY binding and glycosylase activities were assayed as previously described by Lu *et al *[21]. The MutY binding reaction mixture contained 20 mM Tris-HCl, pH7.6, 80 mM NaCl, 1 mM dithiothreitol (DTT), 1 mM EDTA, 2.9% glycerol, 20 ng of poly dI/dC, and 1.8 fmol of labeled DNA (0.09 nM) in 20 μl reactions. After 30 min incubation at 30°C, 3 μl of 50% glycerol was added to the mixtures, and the samples were loaded to a 4% polyacrylamide gel in TBE buffer [50 mM Tris-borate (pH 8.3) and 1 mM EDTA]. After electrophoresis, the gel was dried and exposed to a PhosphorImager screen. Enzyme-bound and free DNA bands were quantified on PhosphorImager and analyzed by ImageQuant (GE Health). To determine active site concentrations, binding experiments were performed with 8 nM of A/GO-containing DNA for wild-type, V45A, and Q182L EcMutY while 30 nM of the same DNA was tested for the V45A/Q182L mutant. The MutY concentrations used ranged from 2 to 400 nM as determined by the Bradford assay.

To determine the *K*_*d *_values, initially, wide ranges of enzyme concentrations with 5-fold series dilution were tested and then nine different enzyme concentrations with 2-fold series dilution (four above and four below the estimated *K*_*d*_) were used. The experiments were repeated at least three times. The *K*_*d *_values were determined under condition of ligand depletion by computer-fitted curve generated by Graph Pad Prism version 3.03 (Graph Pad Software, Inc) using **Eq.1**. The fraction of DNA bound to protein (*f*_*bound*_) was plotted as a function of total active protein concentration in each binding reaction ([P]_*active*_). These data sets were then resolved by nonlinear regression using equation 1, which incorporates ligand depletion and returns the maximal binding fraction (*f*_*max*_) and the equilibrium dissociation constant (*K*_*d*_). Fractional binding in the absence of protein (*f*_*min*_) was set to 0 and the concentration of DNA competent for binding in each reaction is given by [DNA]_T_.

(1)

where *b *= *K*_d _+ [DNA]_T _+ [P]_*active*_.

For *K*_*d *_values without ligand depletion, **Eq. 2 **was used. Under conditions where [DNA]_T _<<*K*_d _(and hence [P]_*active*-*total *_≈ [P]_*active*-*free*_), plots of *f*_*bound *_versus [P]_*active *_were then resolved by nonlinear regression using equation 2.

(2)

The glycosylase assay was carried out in a 10 μl reaction containing 1.8 fmol of DNA substrate (0.18 nM), 20 mM Tris-HCl (pH 7.6), 1 mM DTT, 1 mM EDTA, 2.9% glycerol and 50 μg/ml of bovine serum albumin. After incubation at indicated temperature for 30 min, reaction mixtures were supplemented with 1 μl of 1 M NaOH and heated at 90°C for 30 min. Five μl of formamide dye (90% formamide, 10 mM EDTA, 0.1% xylene cyanol, and 0.1% bromophenol blue) was added to the sample and 5 μl from this mixture was loaded onto a 14% polyacrylamide sequencing gel containing 7 M urea. For time course studies, enzyme reaction was initiated at 37°C or at 4°C for A/G or A/GO-containing DNA, respectively, and aliquot taken at different time points were immediately frozen in -70°C in the presence of 0.1 N NaOH, followed by heating at 90°C for 30 min before adding 5 μl formamide dye and loading to 14% 7 M urea sequencing gels. Bands corresponding to cleavage products and intact DNA were quantified from PhosphorImager images. Graphs and rate constants of glycosylase activities are generated by SigmaPlot for Windows Version 10.0 (Systat Software, Inc.).

### Sequencing of the *rpoB* gene

*E. coli *chromosomal DNA was isolated using a genomic DNA purification kit (Gentra System, Minneapolis, MN). The main group of mutations (cluster II) of the *rpoB *gene was PCR amplified using primers Chang440 (5'-CGTCGTATCCGTTCCGTTGG-3') and Chang441 (5'-TTCACCCGGATACATCTC GTC-3') as described and designed previously [25]. The PCR product was purified with the QIAquick PCR purification kit (QIAGEN, Valencia, CA) and sequenced directly with Chang442 primer (5'-CGTGTAGAGCGTGCGGTGAAA-3').

## Abbreviations

**8-oxoG **or **GO**: 7,8-dihydro-8-oxo-guanines; **DTT**: dithiothreitol; **EcMutY**: *Escherichia coli *MutY; **HhH**: helix-hairpin-helix; **IPTG**: isopropyl β-D-thiogalactoside; ***k*_2_**: rate constants; ***K*_*d*_**: dissociation constant.

## Authors' contributions

AL conceived the study with the participation of PC and AM in the experimental design. PC carried out most of the experiments and initial writing of the manuscript. AM performed the active site titration experiments, analyzed the dissociation constants, and edited the paper. AL revised the manuscript that is then confirmed and approved by other authors.
